# Structural basis for the inhibition of *Mycobacterium tuberculosis* l,d-transpeptidase by meropenem, a drug effective against extensively drug-resistant strains

**DOI:** 10.1107/S0907444912048998

**Published:** 2013-02-16

**Authors:** Hyoun Sook Kim, Jieun Kim, Ha Na Im, Ji Young Yoon, Doo Ri An, Hye Jin Yoon, Jin Young Kim, Hye Kyeoung Min, Soon-Jong Kim, Jae Young Lee, Byung Woo Han, Se Won Suh

**Affiliations:** a Department of Chemistry, College of Natural Sciences, Seoul National University, Seoul 151-742, Republic of Korea; b Research Institute of Pharmaceutical Sciences, College of Pharmacy, Seoul National University, Seoul 151-742, Republic of Korea; c Department of Biophysics and Chemical Biology, College of Natural Sciences, Seoul National University, Seoul 151-742, Republic of Korea; d Division of Mass Spectrometry, Korea Basic Science Institute, Ochang-eup Yeongudangiro 162, Cheongwon-gun, Chungbuk 363-883, Republic of Korea; e Department of Chemistry, Mokpo National University, Chonnam 534-729, Republic of Korea; f Department of Life Science, Dongguk University Seoul, Seoul 100-712, Republic of Korea

**Keywords:** Rv2518c, Mt2594, Ldt_Mt2_, l,d-transpeptidases, *Mycobacterium tuberculosis*, meropenem, carbapenem, peptidoglycans, antituberculosis drug discovery

## Abstract

Difficulty in the treatment of tuberculosis and growing drug resistance in *Mycobacterium tuberculosis* (*Mtb*) are a global health issue. Carbapenems inactivate l,d-transpeptidases; meropenem, when administered with clavulanate, showed *in vivo* activity against extensively drug-resistant *Mtb* strains. Ldt_Mt2_ (Rv2518c), one of two functional l,d-transpeptidases in *Mtb*, is predominantly expressed over Ldt_Mt1_ (Rv0116c). Here, the crystal structure of N-terminally truncated Ldt_Mt2_ (residues Leu131–Ala408) is reported in both ligand-free and meropenem-bound forms. The structure of meropenem-inhibited Ldt_Mt2_ provides a detailed structural view of the interactions between a carbapenem drug and *Mtb* l,d-transpeptidase. The structures revealed that the catalytic l,d-­transpeptidase domain of Ldt_Mt2_ is preceded by a bacterial immunogloblin-like Big_5 domain and is followed by an extended C-terminal tail that interacts with both domains. Furthermore, it is shown using mass analyses that meropenem acts as a suicide inhibitor of Ldt_Mt2_. Upon acylation of the catalytic Cys354 by meropenem, the ‘active-site lid’ undergoes a large conformational change to partially cover the active site so that the bound meropenem is accessible to the bulk solvent *via* three narrow paths. This work will facilitate structure-guided discovery of l,d-transpeptidase inhibitors as novel antituberculosis drugs against drug-resistant *Mtb*.

## Abstract 2

The crystal structure of *M. tuberculosis* l,d-transpeptidase (Ldt_Mt2_; Rv2518c) has been determined in both ligand-free and meropenem-bound forms. The detailed view of the interactions between meropenem and Ldt_Mt2_ will be useful in structure-guided discovery of new antituberculosis drugs.

## 1. Introduction

*Mycobacterium tuberculosis* (*Mtb*), which is a highly successful intracellular pathogen, infects nearly one-third of the world’s population. It causes tuberculosis (TB), which claims the lives of millions of people every year (Dye & Williams, 2010 ▶). The treatment of TB is difficult and requires many months of taking a combination of several anti-TB drugs. The growing incidence of multidrug-resistant and extensively drug-resistant (XDR) strains of *Mtb* poses a global health problem (Chiang *et al.*, 2010 ▶). The enormous success of *Mtb* is based on three capabilities: (i) its reprogramming of macrophages after primary infection/phagocytosis to prevent its own destruction, (ii) its initiation of the formation of well organized granulomas comprising different immune cells to create a confined environment for the host–pathogen standoff and (iii) its transition into a stage of dormancy (nonreplicative state) by shutting down its own central metabolism and by terminating replication, thereby rendering itself extremely resistant to host defence and drug treatment (Gengenbacher & Kaufmann, 2012 ▶). An important issue to consider in the development of new anti-TB therapeutics is the phenotypic drug resistance of *Mtb* organisms in the nonreplicative state, which are genetically indistinguishable but distinct from actively multiplying *Mtb* organisms. An example of their major differences is the nature of peptidoglycan cross-linking.

The unusual mycolic acid-containing cell wall of *Mtb* accounts for up to 40% of the dry mass of the cell and the degree of peptidoglycan cross-linking is unusually high (∼70–80%; Goffin & Ghuysen, 2002 ▶; Almeida Da Silva & Palomino, 2011 ▶). The peptidoglycan structure of *Mtb* from a stationary-phase culture revealed a high content (80%) of nonclassical 3→3 cross-links generated by l,d-transpeptidation (Lavollay *et al.*, 2008 ▶), whereas the classical 4→3 cross-links are predominantly formed by the d,d-transpeptidase activity of penicillin-binding proteins during the exponential phase of growth (Goffin & Ghuysen, 2002 ▶; Wietzerbin *et al.*, 1974 ▶). l,d-­Transpeptidases and d,d-transpeptidases are unrelated to each other in amino-acid sequence. Among five paralogues of l,d-transpeptidase in *Mtb*, two functional l,d-­transpeptidases, Ldt_Mt1_ (Rv0116c; 251 amino acids) and Ldt_Mt2_ (Rv2518c; MT2594; 408 amino acids), have been shown to generate 3→3 cross-links connecting two *meso*-diaminopimelic acid (*meso*-DAP) residues at the third position of the stem peptides *in vitro* (Lavollay *et al.*, 2008 ▶; Gupta *et al.*, 2010 ▶). Of the two *Mtb* genes that encode functional l,d-transpeptidases, *ldt_Mt2_* is predominantly expressed at an at least tenfold higher level than *ldt_Mt1_* in all phases of growth (Gupta *et al.*, 2010 ▶). Furthermore, the loss of Ldt_Mt2_ leads to altered colony morphology, loss of virulence and increased susceptibility to amoxicillin–clavulanate during the chronic phase of infection, supporting the idea that 3→3 cross-linking by Ldt_Mt2_ is vital to the physiology of the peptidoglycan and is essential for the virulence of *Mtb* in the cheronic phase (Gupta *et al.*, 2010 ▶).

β-Lactam antibiotics are analogues of d-alanyl-d-alanine, which forms the terminal amino-acid residues on the precursor NAM/NAG-peptide subunits of the nascent peptidoglycan layer. The structural similarity between β-lactam antibiotics and d-­alanyl-d-alanine facilitates their binding to the active site of penicillin-binding proteins and their inhibition of the d,d-­tranpeptidase activity. β-Lactams were previously thought to be ineffective against *Mtb* primarily as a consequence of their rapid hydrolysis by the endogenous mycobacterial β-­lactamase (BlaC), which shows no similarity in sequence or structure to l,d-transpeptidases. However, the discovery that clavulanic acid acts as an irreversible inhibitor of β-lactamases has made functional l,d-transpeptidases of *Mtb* an attractive target for the development of drugs against *Mtb* in the dormant state (Labia *et al.*, 1985 ▶). Carbapenems, a specific class of β-lactam antibiotics (Supplementary Fig. S1^1^), have been shown to inactivate l,d-transpeptidases from *Entero­coccus faecium* (Mainardi *et al.*, 2007 ▶; Dubée, Arthur *et al.*, 2012 ▶) and *Mtb* (Ldt_Mt1_; Dubée, Triboulet *et al.*, 2012 ▶). Ldt_Mt2_, the major l,d-­transpeptidase in *Mtb*, is also likely to be a target of carbapenems and is considered to be physiologically more important than Ldt_Mt1_. A combination of meropenem and clavulanate showed *in vivo* activity against XDR strains of *Mtb* (Hugonnet *et al.*, 2009 ▶) as well as against H37Rv strains (Hugonnet *et al.*, 2009 ▶; Veziris *et al.*, 2011 ▶; England *et al.*, 2012 ▶), further suggesting that meropenem, one of the carbapenems, may inhibit *Mtb* Ldt_Mt2_. Despite the potential importance of *Mtb* l,d-transpeptidase as a novel anti-TB drug target, no structural information on the detailed interactions of carbapenems with any *Mtb* l,d-transpeptidase is presently available. After submitting our manuscript, the crystal structure of *Mtb* Ldt_Mt2_ containing a bound peptidoglycan fragment was published (Erdemli *et al.*, 2012 ▶).

Here, we report the crystal structure of an N-terminally truncated *Mtb* Ldt_Mt2_ (Ldt_Mt2Δ130_) that encompasses residues Leu131–Ala408. In this structure, the catalytic l,d-transpeptidase domain (residues Asp251–Val378) is preceded by a bacterial immunoglobulin-like (Ig-like) Big_5 domain (residues His150–Gly250) and followed by an extended C-­terminal tail (residues Asn379–Ala408) that interacts with both domains. We have determined the structure of Ldt_Mt2Δ130_ in both ligand-free and drug-bound forms: (i) the apo form, (ii) a mercury-derivatized ligand-free form and (iii) a meropenem-complexed form. Cys354, His336 and Ser337 form the catalytic triad in the active site of the l,d-transpeptidase domain. In the meropenem-complexed structure meropenem is covalently bound to Cys354, mimicking the acyl-enzyme intermediate, and the carbonyl O atom of the opened β-lactam ring is stabilized by the oxyanion hole. In the ligand-free mercury-derivatized model a winding loop containing a two-stranded β-­sheet which encompasses residues His300–Asp323 (‘the active-site lid’) is in the open conformation. In the open conformation the catalytic cysteine (Cys354) is exposed to the bulk solvent, while His336 and Ser337 are buried. Upon the acylation of Ldt_Mt2_ by meropenem, the active-site lid undergoes a large conformational change and partially covers the catalytic Cys354 so that the bound meropenem is accessible to the bulk solvent *via* three narrow paths. This study provides new structural insights into the irreversible inhibition of *Mtb* Ldt_Mt2_ by meropenem. It will facilitate the structure-based discovery of *Mtb* l,d-transpeptidase inhibitors as a novel anti-TB drugs against drug-resistant *Mtb*.

## 2. Materials and methods

### 2.1. Protein expression and purification

Several constructs [residues 1–408 (full length), 55–408, 131–408 and 153–408] of the Ldt_Mt2_ gene from *Mtb* strain H37Rv were PCR-amplified and cloned into the expression vector pET-21a(+) (Novagen) using *Nde*I and *Xho*I restriction enzymes. The recombinant proteins fused with a hexahistidine-containing tag (LEHHHHHH) at the C-­terminus were overexpressed in *Escherichia coli* Rosetta2 (DE3) pLysS cells using Luria broth culture medium. Protein expression was induced using 0.5 m*M* isopropyl β-d-1-thio­galactopyranoside and the cells were incubated for an additional 20 h at 303 K following growth to mid-log phase at 310 K. All constructs except for 153–408 were expressed in *E. coli*. Only the 131–408 construct (Ldt_Mt2Δ130_) gave well diffracting crystals. The cells were lysed by sonication in buffer *A* (50 m*M* Tris–HCl pH 7.9, 500 m*M* NaCl, 50 m*M* imidazole) containing 5%(*v*/*v*) glycerol and 1 m*M* phenylmethylsulfonyl fluoride. The crude lysate was centrifuged at 36 000*g* for 1 h. The supernatant was applied onto a HiTrap Chelating HP affinity chromatography column (GE Healthcare) previously equilibrated with buffer *A*. Upon elution with a gradient of imidazole in the same buffer, recombinant Ldt_Mt2Δ130_ protein eluted at an imidazole concentration of 120–180 m*M*. The eluted protein was applied onto a HiLoad XK-16 Superdex 200 prep-grade column (GE Healthcare) previously equilibrated with 20 m*M* Tris–HCl pH 7.9, 200 m*M* NaCl.

### 2.2. Crystallization

Fractions containing recombinant Ldt_Mt2Δ130_ were pooled and concentrated to 15.7 mg ml^−1^ (0.50 m*M*) using a YM10 ultrafiltration membrane (Amicon) for crystallization. Crystals were grown by the sitting-drop vapour-diffusion method at 296 K by mixing 1 µl protein solution and 1 µl reservoir solution. Crystals of Ldt_Mt2Δ130_ in the apo form were obtained using a reservoir solution consisting of 50 m*M* calcium chloride, 100 m*M* bis-tris pH 6.5, 30%(*v*/*v*) polyethylene glycol monomethyl ether 550. They grew to approximate dimensions of 0.3 × 0.3 × 0.2 mm within a few days. Crystals of Ldt_Mt2Δ130_ pre-incubated with meropenem (50.2 m*M*) for 30 min were obtained using a reservoir solution consisting of 200 m*M* lithium chloride, 20%(*w*/*v*) polyethylene glycol 3350. They grew to approximate dimensions of 0.2 × 0.2 × 0.1 mm within a few days.

### 2.3. X-ray data collection and phasing

X-ray diffraction data were collected from a crystal of Ldt_Mt2Δ130_ in the apo form using a Quantum 270 CCD detector system (Area Detector Systems Corporation, Poway, California, USA) on beamline NE3A of Photon Factory (PF), Japan. The crystals of apo Ldt_Mt2Δ130_ belonged to space group *C*2, with unit-cell parameters *a* = 135.6, *b* = 58.6, *c* = 40.9 Å, β = 94.4°. One monomer is present in the asymmetric unit, giving a Matthews parameter and solvent fraction of 2.59 Å^3^ Da^−1^ and 52.6%, respectively. To collect anomalous diffraction data, a crystal of apo Ldt_Mt2Δ130_ was dipped for 20 min into 5 µl of a heavy-atom-containing cryoprotectant solution that consisted of 25%(*v*/*v*) glycerol and 20 m*M* ethylmercury thiosalicylate (EMTS) added to the reservoir solution. Single-wavelength anomalous diffraction (SAD) data were collected from the mercury-derivative crystal of Ldt_Mt2Δ130_ at 100 K using a Saturn A200 CCD detector system (Rigaku, Japan) on beamline 26B1 of SPring-8, Japan. The raw data were processed and scaled using the *HKL*-2000 program suite (Otwinowski & Minor, 1997 ▶). The mercury-derivative crystals of Ldt_Mt2Δ130_ belonged to space group *C*2, with unit-cell parameters *a* = 135.7, *b* = 58.4, *c* = 41.0 Å, β = 94.3°. One monomer is present in the asymmetric unit, giving a Matthews parameter and solvent fraction of 2.59 Å^3^ Da^−1^ and 52.6%, respectively. One mercury site was located per Ldt_Mt2Δ130_ monomer and the SAD phases were calculated using the *AutoSol* program from *PHENIX* (Adams *et al.*, 2010 ▶). X-ray diffraction data were collected from a crystal of meropenem-complexed Ldt_Mt2Δ130_ using an ADSC Quantum 270 CCD detector system on beamline BL-­1A of the Photon Factory (PF), Japan. The crystals of meropenem-complexed Ldt_Mt2Δ130_ belonged to space group *P*2_1_2_1_2_1_, with unit-cell parameters *a* = 68.9, *b* = 73.4, *c* = 104.1 Å. Two monomers are present in the asymmetric unit, giving a Matthews parameter and solvent fraction of 2.08 Å^3^ Da^−1^ and 40.9%, respectively. Data-collection and phasing statistics are summarized in Table 1 ▶.

### 2.4. Model building and refinement

The SAD-phased electron-density map of Ldt_Mt2Δ130_ was interpreted using the automatic model-building program *RESOLVE* (Terwilliger, 2003 ▶) to build an initial model. Subsequent model building was performed manually using the program *Coot* (Emsley *et al.*, 2010 ▶). The model of the mercury-derivatized Ldt_Mt2Δ130_ was refined with the program *REFMAC* (Murshudov *et al.*, 2011 ▶), including bulk-solvent correction. 5% of the data were randomly set aside as test data for the calculation of *R*_free_ (Brünger, 1992 ▶). The model of the mercury-derivatized Ldt_Mt2Δ130_ was used to determine the structures of the apo form and the meropenem complex of Ldt_Mt2Δ130_ by molecular replacement. A cross-rotational search followed by a translational search was performed utilizing the program *MOLREP* (Vagin & Teplyakov, 2010 ▶). The stereochemistry of the refined models was evaluated using *MolProbity* (Chen *et al.*, 2010 ▶). Refinement statistics are summarized in Table 1 ▶.

### 2.5. Mass spectrometry

All mass spectra were acquired using a linear ion-trap mass spectrometer (Thermo Scientific, USA). The recombinant Ldt_Mt2Δ130_ protein (51 µ*M*) was incubated with meropenem (5.1 m*M*) dissolved in 10 m*M* Tris–HCl pH 7.9. All samples were injected at 2 µl min^−1^ in 50% acetonitrile containing 0.1% formic acid. The average molecular mass of each protein sample was determined for multiply charged ions of charge states from +18 to +27. The experimental molecular mass of the apoprotein was calculated to be 31 263.8 g mol^−1^, showing a 0.05% error compared with the theoretical mass of 31 246.8 g mol^−1^. All experimental molecular masses showed a similar shift (16–18 Da) compared with the theoretical masses (Supplementary Table S1).

### 2.6. Equilibrium sedimentation

Equilibrium-sedimentation studies were performed in 20 m*M* Tris–HCl buffer pH 7.9 containing 200 m*M* NaCl at 293 K using a Beckman ProteomeLab XL-A analytical ultracentrifuge. Ldt_Mt2Δ130_ samples were measured at 230, 235 and 280 nm at two different speeds (24 000 and 28 000 rev min^−1^) using three different protein concentrations (0.80, 1.60 and 2.40 µ*M*). All measured data can be fitted well to a monomer model and representative results for apo and meropenem-complexed Ldt_Mt2Δ130_ are presented. The Ldt_Mt2Δ130_ protein concentrations were calculated using an ∊_280 nm_ value of 62 910 *M* ^−1^ cm^−1^.

### 2.7. Accession codes

The coordinates and structure factors have been deposited in the Protein Data Bank under accession codes 4gsq, 4gsr and 4gsu for apo, mercury-derivatized and meropenem-complexed Ldt_Mt2Δ130,_ respectively.

## 3. Results and discussion

### 3.1. Overall structure of Ldt_Mt2_

Of the constructs tested, we could only obtain well diffracting crystals of the 131–408 construct (Ldt_Mt2Δ130_). The crystal structure of Ldt_Mt2Δ130_ was solved by SAD phasing (Table 1 ▶). We refined three models of Ldt_Mt2Δ130_: (i) the apo form, (ii) a mercury-derivatized ligand-free form and (iii) a meropenem-complexed form (Table 1 ▶). The apo and mercury-derivatized crystals contained one monomer of Ldt_Mt2Δ130_ per asymmetric unit, whereas the crystals of the meropenem complex contained two monomers (chains *A* and *B*) in the asymmetric unit. In these models, the N-­terminal residues (Leu131–Ala149 in the apo form, Leu131–Thr145 in the mercury derivative and Leu131–Gln140 in the meropenem complex for both chains *A* and *B*) and C-terminal affinity tag (LEHHHHHH) are disordered. In the apo model, the internal region of the polypeptide chain (Ser305–Tyr318) is also disordered. The mercury compound is bound around Phe215 in the EMTS-derivatized crystal. Each chain of the meropenem complex contains one meropenem linked to Cys354 by a thioester bond.

**Figure 1:**
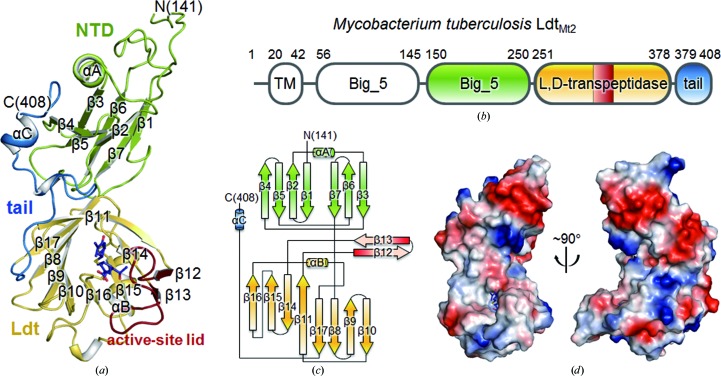
Overall structure of Ldt_Mt2Δ130_. (*a*) Ribbon diagram of meropenem-complexed Ldt_Mt2Δ130_. The NTD and the Ldt domain are shown in green and yellow, respectively. The active-site lid (His300–Asp323) and the C-terminal tail (Asn379–Ala408) are coloured red and blue, respectively. Meropenem bound to the Ldt domain is shown as a stick model. (*b*) Domains of *Mtb* Ldt_Mt2_ coloured as in (*a*). TM, transmembrane helix. (*c*) Topology diagram of Ldt_Mt2Δ130_ coloured as in (*a*). (*d*) Electrostatic surface diagram of meropenem-complexed Ldt_Mt2Δ130_. Blue and red indicate positive and negative electrostatic potentials at neutral pH, respectively.

**Figure 2:**
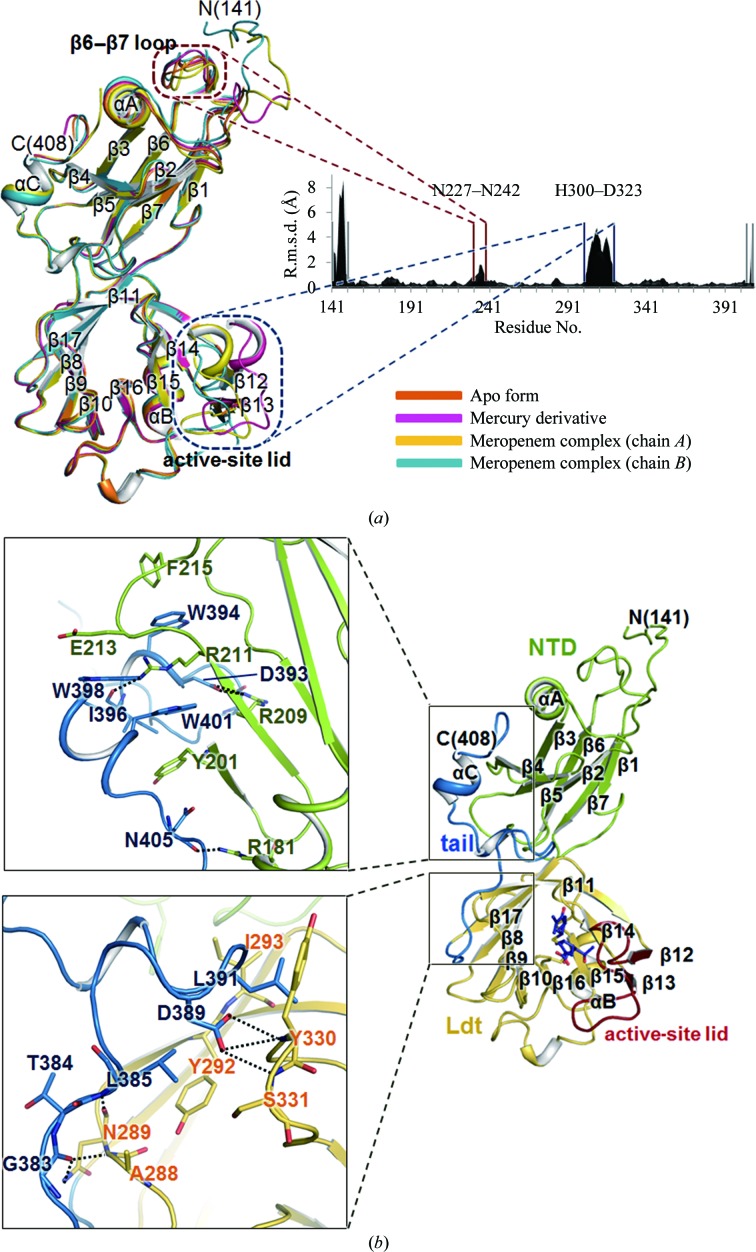
Structural comparisons and domain interactions in Ldt_Mt2Δ130_. (*a*) A superposition of the four chains in the three Ldt_Mt2Δ130_ models and a plot of the C^α^ r.m.s. deviations between any pair of chains averaged over the six pairwise comparisons. The apo, mercury-derivatized and meropenem-complexed Ldt_Mt2_ (chains *A* and *B*) are coloured orange, magenta, yellow and blue, respectively. (*b*) Interactions between the C-terminal tail and two domains coloured as in Fig. 1 ▶(*a*). The enlarged views on the left have slightly different orientations in order to show the detailed interactions better.

Ldt_Mt2Δ130_ is roughly C-shaped, with approximate dimensions of 65 × 45 × 30 Å (Fig. 1 ▶). Ldt_Mt2Δ130_ can be divided into an N-terminal domain (NTD; residues Leu131–Gly250), a catalytic l,d-­transpeptidase (Ldt) domain (residues Asp251–Val378) and a C-terminal tail (residues Asn379–Ala408) (Figs. 1 ▶ *a*–1*c*). The bulk of NTD is folded into a bacterial Ig-like Big_5 domain (His150–Gly250); it comprises a helix (αA) and two antiparallel β-sheets (Fig. 1 ▶). The Ldt domain contains two curved β-­sheets (β16↑–β15↑–β14↓–β11↑ and β11↑–β17↓–β8↓–β9↑–β10↓), with the β11 strand being shared, as well as one α-helix (αB) and two additional antiparallel β-strands (β12↑–β13↓) (Fig. 1 ▶ *c*). In the meropenem-complexed structure (in both chains *A* and *B*), residues His300–Asp323 containing two antiparallel β-strands (β12↑–β13↓) cover the active site of the Ldt domain (‘the active-site lid’), but they are in the open conformation in the mercury-derivatized ligand-free model (Fig. 2 ▶ *a*). The C-terminal tail, which is composed of extended loops and a short α-helix (αC), runs across both the Big_5 and Ldt domains (coloured blue in Figs. 1 ▶ *a*–1*c*).

### 3.2. Structural comparisons and domain interactions in Ldt_Mt2Δ130_

The models of Ldt_Mt2Δ130_ are compared in Fig. 2 ▶(*a*) and the C^α^ root-mean-square (r.m.s.) deviations among them are given in Supplementary Table S2. The r.m.s. deviation between the apo and mercury-derivative models is small (0.40 Å for 246 C^α^-atom pairs), indicating that the mercury binding did not cause a large overall structural change (Supplementary Table S2). However, the r.m.s. deviations between the models of the mercury derivative and of chains *A* and *B* of the meropenem complex are 1.34 and 1.82 Å for 263 C^α^-atom pairs, respectively. Large r.m.s. deviations are observed in the N- and C-­terminal regions (Leu141–His150 and Lys407–Ala408) and two internal regions: the β6–β7 loop of the NTD (Asn227–Asn242) and the active-site lid (residues His300–Asp323) (Fig. 2 ▶ *a*). The C^α^ r.m.s. deviation of the active-site lid is 3.95 Å for chain *B* of the meropenem-complexed model, with a maximum deviation of 6.01 Å occurring at Gly309. The covalent binding of meropenem induces a major conformational rearrangement of the active-site lid from the ‘open’ conformation to the ‘closed’ conformation (Fig. 2 ▶ *a*). The r.m.s. deviation between chains *A* and *B* of the meropenem-complexed model is 1.48 Å for 268 C^α^-atom pairs (Fig. 2 ▶ *a* and Supplementary Table S2) and C^α^ r.m.s. deviations greater than 2.0 Å occur in the four regions mentioned above, with a maximum deviation of 8.89 Å at Pro148. The N-terminal residues around Pro148 are influenced by crystal packing in the meropenem complex. When the ten N-terminal residues (141–150) are excluded from the comparison, the r.m.s. deviation decreases to 0.82 Å, suggesting that the two chains of the meropenem complex represents largely identical states.

The *Protein Interfaces, Surfaces and Assemblies* (*PISA*) server (Krissinel & Henrick, 2007 ▶) revealed that the surface area buried at the interface between the NTD and the Ldt domain is 424, 431, 451 and 450 Å^2^ for the apo form, the mercury derivative and chains *A* and *B* of the meropenem complex, respectively. This indicates that the two domains themselves do not interact extensively with each other. However, the relative orientations of the two domains are virtually identical in all three models of Ldt_Mt2Δ130_ (Fig. 2 ▶ *a*). This is because the C-terminal tail holds them together. The C-­terminal tail (coloured blue in Figs. 1 ▶ *a*–1*c*) interacts with both the Ldt domain and the NTD (Figs. 1 ▶ *a* and 2 ▶ *b*). The αC helix of the C-terminal tail interacts with one face of the large β-­sheet of the NTD *via* hydrogen bonds and salt bridges (Fig. 2 ▶ *b*, upper panel). The aromatic rings of the tryptophan residues (Trp394, Trp398 and Trp401) in the C-terminal tail also stack tightly with the hydrophobic moieties of Tyr201, Arg211, Glu213 and Phe215 of the NTD, further stabilizing the interaction (Fig. 2 ▶ *b*, upper panel). The long β17–αC loop of the C-terminal tail forms a hydrogen-bonding network with the Ldt domain (Fig. 2 ▶ *b*, lower panel).

### 3.3. Analytical ultracentrifugation studies of Ldt_Mt2Δ130_

The largest surface area buried at the interface between two monomers of Ldt_Mt2Δ130_ within the crystals is 501 and 815 Å^2^ per monomer (4.5% and 6.6% of the monomer surface area) for the apo form and the mercury derivative (both in space group *C*2), respectively. Such monomer–monomer contacts can be regarded as crystallization artifacts. On the other hand, the largest surface area buried at the interface between the two tightly packed monomers of the meropenem complex is 1332 Å^2^ per monomer (10.6% of the monomer surface area), suggesting that such a large interface area may exist in solution (Supplementary Fig. S3*a*).

To test whether meropenem binding affects the oligomeric state of Ldt_Mt2Δ130_ in solution, we performed sedimentation-equilibrium studies under various experimental conditions. Supplementary Fig. S3(*b*) shows representative data and fits for monomer (1×) and dimer (2×) models of apo Ldt_Mt2Δ130_. The weighted r.m.s. errors for the monomer (1×) and dimer (2×) fits are 7.93 × 10^−3^ and 4.88 × 10^−2^, respectively, demonstrating the superiority of the 1× model. The residual plots shown in Supplementary Fig. S3(*b*) (top panel) also support our conclusion that apo Ldt_Mt2Δ130_ exists as monomers in solution. In Supplementary Fig. S3(*c*), sedimentation-equilibrium data and fits for the meropenem complex of Ldt_Mt2Δ130_ (after a 1 h reaction with a 100-fold molar excess of meropenem) are shown. The r.m.s. values for the monomer (1×) and dimer (2×) fits are 9.86 × 10^−3^ and 4.79 × 10^−2^, respectively. The r.m.s. values and residual plots (Supplementary Fig. S3*c*, top panel) indicate that meropenem-complexed Ldt_Mt2Δ130_ also exists as monomers in solution. Other heterogeneous or interactive models were also tested, but there was no indication of the possibility of their presence, indicating that meropenem binding has no effect on the monomeric state of Ldt_Mt2Δ130_ in solution. Therefore, we conclude that the apparent dimeric interface observed in the crystal structure of the meropenem complex arises from tight crystal packing.

### 3.4. Ldt_Mt2_ contains tandem immunoglobulin-like Big_5 domains

**Figure 3:**
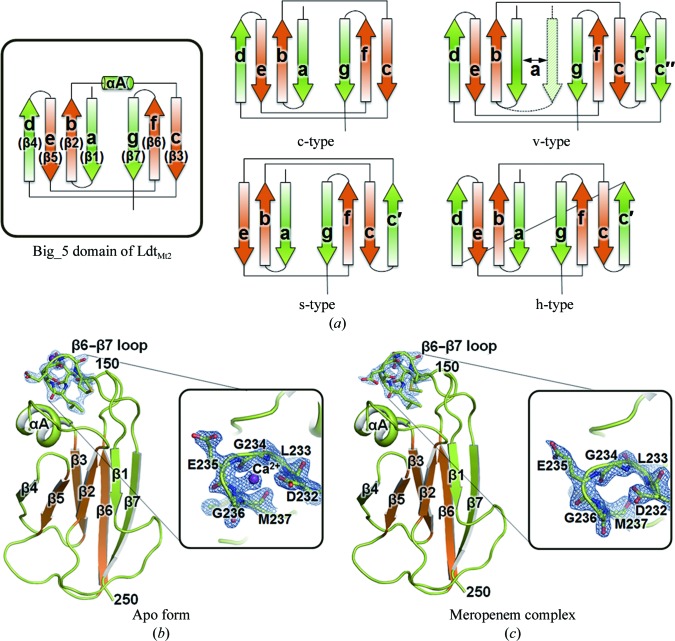
Big_5 domain of Ldt_c__Mt2_. (*a*) Topology diagrams of the Big_5 domain (His150–Gly250) of Ldt_Mt2Δ130_ (inset) and of four distinct subtypes of the Ig-like fold (modified from Bork *et al.*, 1994 ▶): c-type (constant), v-type (variable), s-type (switched) and h-type (hybrid). The four-stranded structural core (strands b, c, e, and f; β2, β3, β5 and β6 of Ldt_Mt2Δ130_) common to all Ig-like domains (orange) is surrounded by structurally more variable strands (green). (*b*, *c*) Ribbon diagrams of the Big_5 domain (His150–Gly250) in the apo model of Ldt_Mt2Δ130_ (*b*) and meropenem-complexed Ldt_Mt2Δ130_ (*c*), and enlarged views of the β6–β7 loop (insets) coloured as in (*a*). The bound calcium ion and the residues around it (Asp232–Met237) are shown as a purple ball and as stick models, respectively, with a 2*mF*_o_ − *DF* electron-density map (contoured at 1.5σ).

Ldt_Mt2_ possesses an additional N-terminal region (residues Met1–Asp251) which contains a putative transmembrane helix (Leu20–Ala42) in front of its catalytic Ldt domain (Fig. 1 ▶ *b*). Sequence analysis using the Prosite database (http://www.expasy.org/prosite) reveals that Ldt_Mt2_ appears to be a putative lipoprotein with Cys35 as a potential lipid-attachment site. Unexpectedly, our crystal structure of Ldt_Mt2Δ130_ reveals that His150–Gly250 of Ldt_Mt2Δ130_ are folded into a compact Ig-­like domain (Fig. 3 ▶ *a*, inset; Bork *et al.*, 1994 ▶), although it shows no detectable sequence similarity to eukaryotic Ig-fold proteins. A *DALI* structural-similarity search (Holm & Rosenström, 2010 ▶) reveals that the NTD of Ldt_Mt2Δ130_ structurally resembles the light chain of antibody Fab fragments such as the *Mus musculus* 6E1 Fab light chain (Jiang *et al.*, 2003 ▶; PDB entry 1orq; r.m.s. deviation of 3.3 Å for 86 equivalent C^α^ positions, a *Z*-score of 7.2 and a sequence identity of 6%).

Ig folds have been identified in prokaryotic proteins and are known as bacterial immunoglobulin-like (Big) domains (Halaby *et al.*, 1999 ▶). Big domains are present in proteins ranging from enzymes to chaperones (Halaby & Mornon, 1998 ▶). Among the topological subtypes of classical Ig-like domains (Bork *et al.*, 1994 ▶), the NTD of Ldt_Mt2Δ130_ follows the topology of the C-type (constant) Ig fold, as the NTD of Ldt_Mt2Δ130_ does not possess an extra loop between strands β3 and β4 (Fig. 3 ▶ *a*). Thus, the Big domain in the NTD of Ldt_Mt2Δ130_ is structurally more similar to the constant region of the immunoglobulin light chain in the Fab fragment than the variable region (Fig. 3 ▶ *a*).

Following our structural analyses, we searched for other YkuD-family proteins that contain one or more Big domains such as Big_2 (Pfam accession No. PF02368), Big_3 (PF07523) or Big_5 (PF13205). The overall amino-acid sequence of Ldt_Mt2_ aligns well with those of proteins containing Big_5 domains, such as a membrane protein from *Streptomyces* sp. SPB74 (ExPASy accession No. B5GI65; overall sequence identity of 29%) and an YkuD-family protein from *Nakamurella multipartita* (ExPASy accession No. C8X6V6; overall sequence identity of 40%). Interestingly, the *N. multipartita* YkuD protein possesses two sequential Big_5 domains: one in residues 182–282 (corresponding to His150–Gly250 of Ldt_Mt2_) and one in residues 87–177 (corresponding to Asp56–Thr145 of Ldt_Mt2_). This clearly indicates that Ldt_Mt2_ also has two sequential Big_5 domains in front of its Ldt domain. In comparison, the minor l,d-transpeptidase in *Mtb*, Ldt_Mt1_, contains only a single Big_5 domain that shows 34% sequence identity to the second Big_5 domain of Ldt_Mt2_ but shows no sequence identity to the first Big_5 domain of Ldt_Mt2_. The first Big_5 domain of Ldt_Mt2_ may not be essential for catalytic activity, as Ldt_Mt1_ has been shown to be functional (Lavollay *et al.*, 2008 ▶; Dubée, Triboulet *et al.*, 2012 ▶). Our Ldt_Mt2Δ130_ structures reveal that the second Big_5 domain is rigidly held by the Ldt domain *via* the C-terminal tail. However, the C-terminal tail does not appear to be sufficiently long to interact with the first Big_5 domain. The C-terminal tail is also conserved in the above two proteins that contain Big_5 domains, suggesting that the observed domain arrangement of Ldt_Mt2Δ130_ may also be conserved and that the tandem Big_5 domains, in particular the second one, may play an important role in assisting the function of the catalytic Ldt domain.

Although the biological function of the Big_5 domains of Ldt_Mt2_ is unknown, various aspects of the general functions of other Big domains have been studied. For example, leptospiral immunoglobulin-like (Lig) proteins, which are surface-exposed proteins that belong to the Big_2 family, mediate host–pathogen interactions (Castiblanco-Valencia *et al.*, 2012 ▶). The Big_2 domains of Lig proteins have been reported to bind Ca^2+^, suggesting possible involvement of Ca^2+^ binding in the function of proteins containing Big domains (Raman *et al.*, 2010 ▶). Our crystals of apo Ldt_Mt2Δ130_ were obtained using a reservoir solution containing 50 m*M* calcium chloride, whereas the crystallization condition for the meropenem complex did not contain calcium ions. Interestingly, we observed extra electron density in close proximity to the β6–­β7 loop in the structure of the apo form (Fig. 3 ▶ *b*), whereas no such electron density was observed at the same position in the meropenem complex (Fig. 3 ▶ *c*). The extra electron density is best interpreted as a Ca^2+^ ion bound to the Big_5 domain because the density peak is in close association with the backbone N atoms of Gly234 and Glu235 at distances of 2.33 and 2.78 Å, respectively, as well as a side-chain O atom of Asp232 at a distance of 2.93 Å (Figs. 3 ▶ *b* and 3 ▶ *c*). Gly234 and Gly236 appear to be crucial in allowing a sharp turn in the β6–­β7 loop, which exhibits large r.m.s. deviations between the apo and meropenem-complex structures (Fig. 2 ▶ *a*). Asp232, Gly234 and Gly236 of Ldt_Mt2_ are semi-conserved in other Big_5-domain-containing proteins as (i) Glu235, Ala237 and Gly239 in the membrane protein from *Streptomyces* sp. SPB74, (ii) Asn264, Gly266 and Gly268 in the YkuD-family protein from *N. multipartita* and (iii) Glu73, Thr75 and Gly77 in another functional *Mtb* l,d-transpeptidase, Ldt_Mt1_.

### 3.5. l,d-Transpeptidase domain of Ldt_Mt2_, its covalent acylation by meropenem and a structural view

Our structures of Ldt_Mt2Δ130_ show that the C-terminal region of Ldt_Mt2_ (residues Asp251–Val378) is folded into a compact domain with the l,d-transpeptidase fold. Previously reported structural data on l,d-transpeptidases include the crystal structure of a C-terminal fragment (Ldt_fm_; residues 217–466) of *E. faecium* l,d-transpeptidase in the apo form (Biarrotte-Sorin *et al.*, 2006 ▶), the crystal structure of the Lys117Ala/Gln118Ala double mutant of *Bacillus subtilis* YkuD protein (Ldt_Bs_) in the apo form (Bielnicki *et al.*, 2006 ▶) and the solution structure of Ldt_Bs_ in the apo and imipenem-bound forms (Lecoq *et al.*, 2012 ▶). As expected, the two most similar structures detected using the *DALI* program (Holm & Rosenström, 2010 ▶) are (i) Ldt_Bs_ (Bielnicki *et al.*, 2006 ▶; PDB entry 1y7m; *Z*-­score of 17.7, r.m.s. deviation of 4.3 Å and sequence identity of 17% for 131 equivalent C^α^ positions) and (ii) Ldt_fm_ (Biarrotte-Sorin *et al.*, 2006 ▶; PDB code 1zat, *Z*-score of 16.8, r.m.s. deviation of 6.5 Å and sequence identity of 20% for 151 equivalent C^α^ positions). The *Z*-scores are below 5 for other proteins. The Ldt_fm_ structure consists of a domain (residues 217–336) with a novel mixed α/β fold and the l,d-­transpeptidase catalytic domain (residues 337–466). Ldt_Bs_ is encoded by the *ykuD* gene of *B. subtilis* and is one of three putative l,d-transpeptidases. Its l,d-transpeptidase catalytic domain (residues 55–164) is preceded by a LysM domain (residues 4–46). In the solution structure of imipenem-bound Ldt_Bs_ the covalently bound imipenem ligand samples a wide range of orientations and does not adopt a well defined conformation, thus providing no detailed view of the ligand–protein interactions (Lecoq *et al.*, 2012 ▶; De & McIntosh, 2012 ▶). Previously, it has been shown that the soluble fragment (residues Ala55–Ala408) of Ldt_Mt2_ containing the l,d-transpeptidase catalytic domain (residues Asp251–Val378) catalyzes the formation of 3→3 peptidoglycan cross-links with disaccharide–tetrapeptide monomers *in vitro* (Gupta *et al.*, 2010 ▶). It has been shown that the l,d-transpeptidase activities of Ldt_Mt2_ and Ldt_Mt1_ can be inactivated by carbapenems, such as meropenem, which is effective against drug-resistant *Mtb* strains (Dubée, Triboulet *et al.*, 2012 ▶; Hugonnet *et al.*, 2009 ▶).

Using mass spectrometry, we observed the covalent binding of meropenem to Ldt_Mt2Δ130_ (Supplementary Fig. S4 and Table S1). After 20 min of incubating Ldt_Mt2Δ130_ with meropenem, both a first set of peaks corresponding to the mass of the covalently acylated Ldt_Mt2Δ130_–meropenem complex and a second set of peaks which were 44 Da smaller than the first set of peaks were present (Supplementary Figs. S4*a* and S4*b* and Table S1). After 3 d of incubation, only the second set of peaks remained (Supplementary Figs. S4*a* and S4*c* and Table S1). The covalent meropenem adduct corresponding to the second set of peaks could be produced by the subtraction of an acetaldehyde moiety (*m*/*z* = 44 Da), similarly to the previously proposed mechanism for meropenem-bound β-lactamase from *Mtb* (Hugonnet *et al.*, 2009 ▶). These experiments indicate that meropenem-acylated Ldt_Mt2Δ130_ is stable for at least 3 d after β-lactam ring opening, demonstrating that the deacylation of meropenem-inactivated Ldt_Mt2Δ130_ is slow. In the case of *Mtb* Ldt_Mt1_, the hydrolysis rate was 3100 times slower than the acylation rate (Dubée, Triboulet *et al.*, 2012 ▶).

To provide a detailed structural view of how meropenem inhibits Ldt_Mt2_, we have determined the crystal structure of Ldt_Mt2Δ130_ as a covalent complex with meropenem. In chain *B* of the meropenem-complex model, clear electron density for meropenem that is contiguous with the S^γ^ atom of Cys354 was observed (Supplementary Fig. S2), but the electron density was missing beyond the thioether S atom of meropenem. This is likely to reflect the free orientation of the terminal region of meropenem corresponding to the *R* ^3^ side chain of carbapenem (Supplementary Figs. S1 and S2). In chain *A* the covalently bound meropenem is observed up to the pyrroline ring and is less clear than in chain *B* (Supplementary Fig. S2). Therefore, we restrict our detailed discussion of meropenem–Ldt_Mt2Δ130_ interactions to chain *B* only.

**Figure 4:**
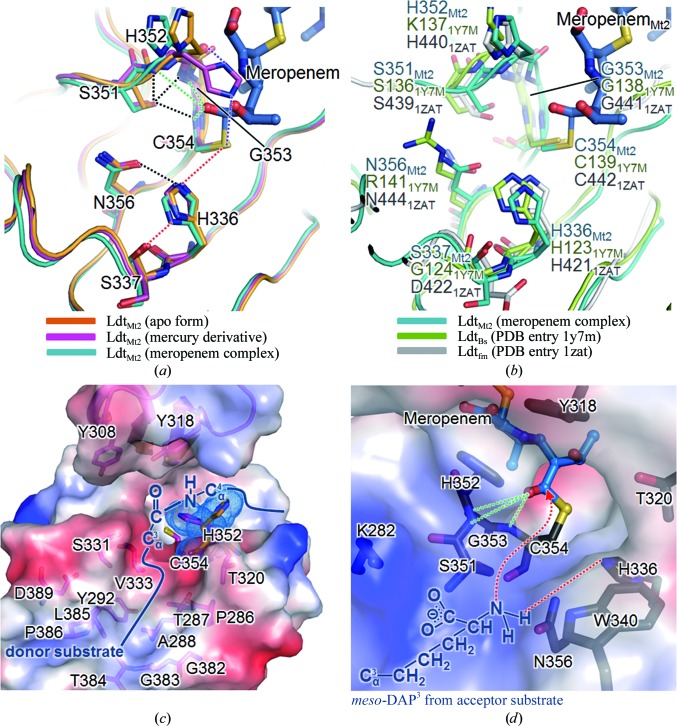
The active site of the Ldt domain and the substrate-binding sites. (*a*) Active-site superposition of the apo (orange), mercury-derivatized (magenta) and meropenem-complexed (chain *B*, cyan) Ldt_Mt2Δ130_. Dotted lines denote interactions: hydrogen bonds to the catalytic triad (red) and the oxyanion hole (green), Ser351 with the oxyanion hole (black), His336 with Asn356 (black) and His352 with Cys354 S^γ^ and the main-chain carbonyl O atom of His352 in the mercury-derivatized model (purple). The covalently bound meropenem adduct in the meropenem complex is shown as a stick model. (*b*) Active-site superposition of meropenem-complexed Ldt_Mt2_ (chain *B*, cyan), Ldt_Bs_ (green) and Ldt_fm_ (grey) in the same view as in (*a*). (*c*, *d*) Electrostatic surface representations of the predicted binding sites for the donor substrate in the open conformation of mercury-derivatized Ldt_Mt2Δ130_ (*c*) and the acceptor substrate in the closed conformation of meropenem-complexed Ldt_Mt2Δ130_ (*d*) coloured as in Fig. 1 ▶(*d*). The surfaces of the binding site are represented with constituent residues as stick models. The movement of the imidazole ring of His352 in the apo form (orange) and the mercury-derivatized form (magenta) is presented in stick models with dotted surfaces in (*c*). The peptide bond of the donor substrate (*meso*-DAP^3^-d-Ala^4^) is schematically modelled into the active site in the open conformation. The terminus of the acceptor substrate (*meso*-DAP^3^) is schematically modelled into the active site in the closed conformation, with red dotted lines depicting a plausible site for recognizing the terminal amine of *meso*-DAP^3^.

The invariant Cys354 of Ldt_Mt2_ has been proposed to be the key catalytic residue in l,d-transpeptidase activity (Supplementary Fig. S5; Lecoq *et al.*, 2012 ▶). Furthermore, we note that the disposition of three residues, His336 and Ser337 (both located on β15) and Cys354 (located on β16), is highly similar to the catalytic triad of a wide range of enzymes including serine proteases, esterases and β-lactamases (Fig. 4 ▶ *a*; Dodson & Wlodawer, 1998 ▶). Similar catalytic triads, **H**(S/G/D)*x*_16–19_ **C**, are also present in the Ldt_Bs_ and Ldt_fm_ structures (Fig. 4 ▶ *b* and Supplementary Fig. S5), where *x* represents any amino acid and the strictly conserved residues are shown in bold. The main-chain carbonyl of Ser337 in Ldt_Mt2_, as well as the corresponding Gly127 in Ldt_Bs_ and Asp422 in Ldt_fm_, acts as a hydrogen-bond acceptor. In our three structures of Ldt_Mt2Δ130_, the side chains of His336 and Cys354 are suitably positioned for a cooperative action with the main-chain carbonyl of Ser337 (marked by dashed red lines in Fig. 4 ▶ *a*). The N^∊2^ atom of His336 interacts with the S^γ^ atom of Cys354 with a distance of 4.09 Å, and the N^δ1^ atom of His336 interacts with the main-chain carbonyl O atom of Ser337 with a distance of 2.86 Å. The side-chain conformation of the catalytic His336 is stabilized by an additional interaction between His336 N^∊2^ and Asn356 O^δ1^ with a distance of 3.37 Å (Fig. 4 ▶ *a*).

The structure of the meropenem complex of Ldt_Mt2_ represents the acylated-enzyme intermediate state (Fig. 4 ▶ *a*) in the proposed mechanism of l,d-transpeptidation (Supplementary Fig. S6) based on our structures and previous studies (Dodson & Wlodawer, 1998 ▶; Lecoq *et al.*, 2012 ▶). In analogy with the proposed mechanism for Ldt_Bs_ (Lecoq *et al.*, 2012 ▶), we propose that His336 captures the S^γ^ proton of Cys354 to generate the nucleophilic thiolate that attacks the carbonyl C atom of the meropenem β-lactam ring, resulting in an acylated enzyme; His336 then releases its acidic proton to the N atom of the β-lactam ring (Supplementary Fig. S6). Upon nucleophilic attack by the thiolate of Cys354, the carbonyl C atom of the β-lactam ring of meropenem is covalently bound to the S^γ^ atom of Cys354. The bond distance between Cys354 S^γ^ and the carbonyl C atom of meropenem is 1.94 Å, which falls within the typical bond distances (1.81–2.55 Å) of carbon–sulfur single bonds (Fig. 4 ▶).

The so-called oxyanion hole stabilizes a tetrahedral enzyme–substrate intermediate (E-S_1_* in Supplementary Fig. S6) to increase the activity of l,d-transpeptidation. In Ldt_Mt2_, His352, Gly353 and Cys354 contribute to the formation of the oxyanion hole, in which the carbonyl group of the opened meropenem scissile bond is hydrogen-bonded to the backbone amide N atoms of His352, Gly353 and Cys354 with distances of 3.83, 3.27 and 2.83 Å, respectively (Fig. 4 ▶ *a* and Supplementary Fig. S6).

### 3.6. The active-site lid undergoes a large conformational change upon acylation by meropenem

**Figure 5:**
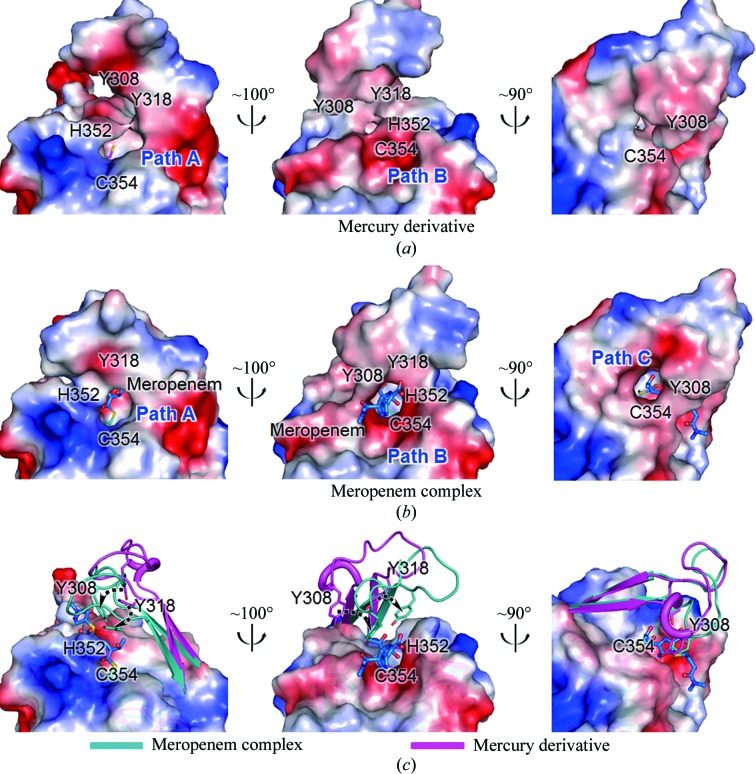
Conformational flexibility of the active-site lid in Ldt_Mt2Δ130_. (*a*, *b*) Three different views of the electrostatic potential surface diagrams of mercury-derivatized (*a*) and meropenem-complexed Ldt_Mt2Δ130_ (*b*). The closed conformation of the active-site lid in (*b*) reveals that meropem attached to catalytic Cys354 is accessible through three narrow paths (Paths A, B and C). (*c*) The active-site lids in meropenem-complexed (cyan) and mercury-derivatized (magenta) Ldt_Mt2Δ130_ are shown as ribbon models with the surface of the meropenem complex. Residues that show large shifts upon lid closure are shown as stick models; the movement is indicated by black dotted arrows. The two models are in the same orientation.

The active-site lid (His300–Asp323) of *Mtb* Ldt_Mt2_ is much longer than those of Ldt_Bs_ and Ldt_fm_ (Supplementary Fig. S5). The active-site lid of *Mtb* Ldt_Mt1_ is equally long. Those of Ldt_Bs_ and Ldt_fm_ are truncated and appear to be too short to fully cover the active-site pocket of their l,d-transpeptidase domains. A comparison of our Ldt_Mt2Δ130_ structures demonstrates the conformational flexibility of the active-site lid (Figs. 2 ▶ *a* and 5 ▶). The active-site lid adopts an open conformation in the mercury-derivative structure (Fig. 5 ▶ *b*), whereas it is in a closed conformation in the meropenem complex (Fig. 5 ▶ *a*). This observation suggests that the open active-site lid before acylation by meropenem undergoes movement to close the active-site pocket upon acylation (Fig. 5 ▶ *c*). The flexible nature of the active-site lid is further supported by the apo structure, in which much of the lid is disordered. Upon closure of the active-site lid, the side chains of Tyr308 and Tyr318 move close to the active-site pocket (Fig. 5 ▶ *c*), with their O^η^ atoms shifting by 5.46 and 4.70 Å, respectively. The two tyrosines enclose tightly the covalently bound meropenem (Figs. 5 ▶ *a* and 6 ▶ *b*–6*d*).

In the open conformation before acylation, the S^γ^ atom of Cys354 is exposed to bulk solvent and appears to be readily accessible *via* two wide paths (Paths A and B in Fig. 5 ▶ *a*). In the closed conformation of the meropenem complex, meropenem attached covalently to Cys354 is accessible from the bulk solvent along three narrower paths which are formed by the closure of the active-site lid (Paths A, B and C in Fig. 5 ▶ *b*). Path B is occupied by the *R* ^3^ part of meropenem, indicating that Path B is likely to be the binding site for the donor substrate (S_1_; Fig. 4 ▶ *c*). The S^γ^ atom of Cys354 points toward the entrance to Path B in our three structures, suggesting that the *meso*-DAP^3^ part of the donor substrate (the peptide bond of *meso*-DAP^3^-d-Ala^4^; S_1_) may approach Cys354 along Path B (Fig. 4 ▶ *c* and ‘E + S_1_’ in Supplementary Fig. S6).

Moreover, the amine group of the side chain of the second substrate (*meso*-DAP^3^ of the acceptor peptide; S_2_) is expected to approach His336 of the catalytic triad to form a tetrahedral enzyme–substrate intermediate (‘AcylE+S_2_’ and ‘AcylE-S_2_*’ in Supplementary Fig. S6 and Fig. 4 ▶ *d*). Therefore, approach of the acceptor substrate for transpeptidation seems to only be allowed through Path A, in which the acylated S^γ^ of Cys354 is exposed and His336 lies on the surface of Path A (Figs. 4 ▶ *d* and 5 ▶ *a*). In addition, the surface of Path A is lined by Gly281, Lys282, Trp340 and Asn356 (Fig. 4 ▶ *d*). Gly281 and Lys282 are strictly conserved in the YkuD family, while Trp340 and Asn356 are semi-conserved (Supplementary Fig. S5). The strict conservation of Gly281 and Lys282 (boxed in brown in Supplementary Fig. S5) might be related to the possibility that the positive charge of Lys282 stabilizes the carboxyl group of the side chain of the acceptor substrate (S_2_) *meso*-DAP^3^ (Fig. 4 ▶ *d*). The invariant Lys282 of *Mtb* Ldt_Mt2_ may be critical in recognizing the acceptor peptide during the transpeptidation reaction that is catalyzed by l,d-transpeptidase (Supplementary Fig. S6).

### 3.7. Implications for anti-TB drug design

**Figure 6:**
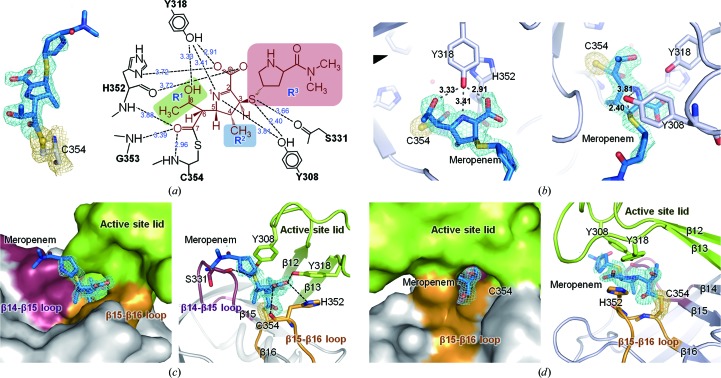
Meropenem-inactivated Ldt_Mt2Δ130_. (*a*) Electron-density map (left) and a schematic diagram of interactions (right) of the covalently bound meropenem adduct with Cys354 in meropenem-complexed Ldt_Mt2Δ130_ (chain *B*). The OMIT *mF*_o_ − *DF*_c_ map (contoured at 2.5σ) for meropenem and the 2*mF*_o_ − *DF*_c_ map (contoured at 1.0σ) for Cys354 are coloured blue and yellow, respectively. Dotted lines denote interactions with Ldt_Mt2Δ130_ and the corresponding bond lengths are shown in Å. Variable regions (*R* ^1^, *R* ^2^ and *R* ^3^) of carbapenems are shaded in green, blue and red, respectively. (*b*) Interactions of the bound meropenem with Tyr318 (left) and Tyr308 (right). Dotted lines and the electron-density map are presented as in (*a*). (*c*) Surface representation (left) and ribbon diagram (right) of the active site of Ldt_Mt2Δ130_ enclosing the meropenem adduct viewed along Path B. The β14–β15 loop, the β15–β16 loop and the active-site lid, which surround the bound meropenem, are coloured plum, orange and green, respectively. (*d*) Surface representation (left) and ribbon diagram (right) of the active site in the meropenem-complexed Ldt_Mt2Δ130_ viewed along Path A presented as in (*c*).

Our mass-spectrometric and crystal structure analyses revealed that meropenem is covalently bonded to the S^γ^ atom of Cys354 of *Mtb* Ldt_Mt2_. We have also shown that Ldt_Mt2_ is not recovered from the meropenem-inactivated acyl enzyme for at least 3 d after the acylation reaction. Even though it took three weeks from initial incubation with meropenem to the collection of X-ray diffraction data, the electron-density map clearly indicated the covalently bonded meropenem adduct, supporting the ability of meropenem to act as a suicide inhibitor of Ldt_Mt2_. Our structure of meropenem-acylated Ldt_Mt2_ sheds light on the design of improved anti-TB carbapenems by providing a detailed view of the interactions between meropenem and Ldt_Mt2_ (Fig. 6 ▶).

The meropenem C7 carbonyl group is bonded to Cys354 and forms hydrogen bonds to the backbone N atoms of His352, Gly353 and Cys354 in the oxyanion hole in chain *B* of the meropenem-inhibited model (Fig. 6 ▶ *a*). The side chains of two tyrosine residues, Tyr308 and Tyr318, from the active-site lid make hydrogen bonds to meropenem (Fig. 6 ▶ *b*). The ζ-­hydroxyl group of Tyr308 interacts with the meropenem N1 atom and the thioether S atom of meropenem C3. In addition, the pyrroline ring of meropenem is stabilized by the aromatic ring of Tyr308 through hydrophobic interactions. The highly conserved Tyr318 interacts with His352 by π-stacking and forms hydrogen bonds to the C8 hydroxyl group, the N1 atom and the C2 carboxyl group of meropenem (Figs. 6 ▶ *a* and 6 ▶ *b*). These protein–meropenem interactions indicate that the two tyrosine side chains may undergo a large shift in their positions upon acylation by meropenem, thus triggering the observed conformational change in the active-site lid. The backbone carbonyl groups of Ser331 and His352, as well as the side chain of His352, also make hydrogen bonds to the bound meropenem.

Much of the *R* ^3^ part of meropenem beyond the thioether S atom (Supplementary Fig. S1) does not make tight inter­actions with the protein. This observation explains why the electron density is missing beyond the thioether S atom of the opened meropenem (for chain *B*). The surface of the potential binding site for the donor substrate (S_1_) or the *R* ^3^ side chain of carbapenem is mainly lined by hydrophobic residues, except for the hydroxyl groups from the side chains of Ser331, Thr287 and Tyr292 (Fig. 4 ▶ *c*). Based on the surface analysis of the meropenem-bound form, it appears that the *R* ^3^ side chain of carbapenems can be tailored to better accommodate the protein surface and to optimize their inhibitory activities towards Ldt_Mt2_ (Supplementary Fig. S7). This view is in agreement with previous findings that the *R* ^3^ side chain of the carbapenem modulates the kinetic constants for the inactivation reaction of Ldt_Mt1_ (Dubée, Triboulet *et al.*, 2012 ▶) and Ldt_fm_ (Dubée, Arthur *et al.*, 2012 ▶). Moreover, inhibitor design towards the potential binding site of the second substrate (*meso*-DAP^3^ of the acceptor peptide; S_2_) could also be attempted, thereby blocking the final transpeptidation step of 3→3 cross-linking. The surface representation of the potential binding site for the acceptor substrate (S_2_; Fig. 4 ▶ *d*) provides the semi-conserved target residues, *e.g.* Lys282, Trp340 and Asn356, for the targeted inhibition of the transpeptidation step. As the key residues in the active sites of Ldt_Mt2_ and Ldt_Mt1_ are well conserved (Supplementary Fig. S5), a designed inhibitor is likely to inhibit both.

## 4. Conclusions

This study provides the first detailed structural view of the meropenem-inactivated l,d-transpeptidase domain of Ldt_Mt2_, which is predominantly expressed by *Mtb*. The l,d-transpeptidase domain of the minor l,d-transpeptidase in *Mtb*, Ldt_Mt1_, is expected to be highly similar in its structure as it shows 48% sequence identity and the key catalytic residues are conserved. Together with the previous kinetic studies (Dubée, Triboulet *et al.*, 2012 ▶), our structural and mass-spectrometric analyses of the meropenem complex indicate that Ldt_Mt2_ is not readily recovered from the meropenem-acylated form. A large conformational change in the active-site lid of Ldt_Mt2_ upon acylation by meropenem to partially cover the bound meropenem may explain the long-lived acylated enzyme intermediate. This structural information will be valuable in further optimization of meropenem and will facilitate the structure-based discovery of new anti-TB carbapenem drugs.

## Supplementary Material

PDB reference: Ldt_Mt2_, apo, 4gsq

PDB reference: mercury derivative, 4gsr

PDB reference: meropenem complex, 4gsu

Supplementary material file. DOI: 10.1107/S0907444912048998/be5223sup1.pdf

## Figures and Tables

**Table 1:** Data-collection and refinement statistics Values in parentheses are for the highest resolution shell.

Data set	Apo form	Hg derivative (peak)	Meropenem complex
Data collection			
Space group	*C*2	*C*2	*P*2_1_2_1_2_1_
Unit-cell parameters			
*a* (Å)	135.6	135.7	68.9
*b* (Å)	58.6	58.4	73.4
*c* (Å)	40.9	41.0	104.1
α = γ (°)	90.0	90.0	90.0
β (°)	94.4	94.3	90.0
X-ray wavelength (Å)	1.00000	1.00600	1.00000
Resolution range (Å)	20.0–1.80 (1.83–1.80)	50.0–1.79 (1.82–1.79)	50.0–2.00 (2.03–2.00)
Total No. of reflections	114133 (3344)	218163 (7927)†	254969 (11833)
No. of unique reflections	29426 (1045)	58915 (2557)†	34693 (1621)
Completeness (%)	97.9 (70.4)	99.3 (86.8)†	95.5 (91.2)
〈*I*/σ(*I*)〉	46.5 (9.1)	43.0 (7.4)†	31.0 (4.3)
*R*_merge_ ‡ (%)	4.6 (14.4)	4.6 (14.9)†	12.8 (89.1)
SAD phasing			
Figure of merit (before/after density modification)			0.41/0.60
Model refinement			
PDB code	4gsq	4gsr	4gsu
Resolution range (Å)	20.0–1.80	20.0–1.79	20.0–2.00
*R*_work_/*R*_free_ § (%)	19.7/23.3	19.6/22.6	18.9/23.2
No. of non-H atoms/average *B* factor (Å^2^)			
Protein	1889/23.5	2014/23.1	4112/26.4
Water oxygen	206/32.9	230/36.3	228/33.0
Meropenem	—	—	52/65.5
EMTS	—	11/38.0	—
Glycerol	6/53.3	12/44.1	—
Calcium ion	1/65.2	—	—
Wilson *B* factor (Å^2^)	21.9	20.4	23.5
R.m.s. deviations from ideal geometry			
Bond lengths (Å)	0.009	0.007	0.010
Bond angles (°)	1.29	1.15	1.40
R.m.s. *Z*-scores¶			
Bond lengths	0.450	0.335	0.491
Bond angles	0.590	0.512	0.622
Ramachandran plot†† (%)			
Favoured/outliers	98.8/0.0	98.5/0.0	97.9/0.2
Poor rotamers†† (%)	1.00	0.47	0.91

† Friedel pairs were treated as separate observations.

‡ *R*_merge_ = 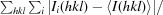 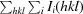, where *I*(*hkl*) is the intensity of reflection *hkl*, 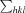 is the sum over all reflections and 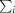 is the sum over *i* measurements of reflection *hkl*.

§ *R*_work_ = 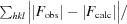 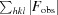, where *R*_free_ is calculated for a randomly chosen 5% of reflections which were not used for structure refinement and *R*_work_ is calculated for the remaining reflections.

¶ Values obtained using *REFMAC*.

†† Values obtained using *MolProbity*.

## Footnotes

1 Supplementary material has been deposited in the IUCr electronic archive (Reference: BE5223). Services for accessing this material are described at the back of the journal.
