# Supplementary material for: Structural basis for the inhibition of Mycobacterium tuberculosis l,d-transpeptidase by meropenem, a drug effective against extensively drug-resistant strains
Source: Acta Crystallogr D Biol Crystallogr. 2013 Feb 16;69(Pt 3):420–31. doi: 10.1107/S0907444912048998 (PMC3605043; doi:10.1107/S0907444912048998)
Supplement: Supplementary file 1 [file d-69-00420-sup1.pdf]

Supplementary Information for

Structural basis for the inhibition of *Mycobacterium tuberculosis* L,D-transpeptidase by meropenem, a drug effective against extensively drug-resistant strains

Hyoun Sook Kim[a,b], Jieun Kim[c], Ha Na Im[c], Ji Young Yoon[a], Doo Ri An[c], Hye Jin Yoon[a], Jin Young Kim[d], Hye Kyeoung Min[d], Soon-Jong Kim[e], Jae Young Lee[f], Byung Woo Han[b], and Se Won Suh[a,c]*

[a]Department of Chemistry, College of Natural Sciences, Seoul National University, Seoul 151-742, Korea, [b]Research Institute of Pharmaceutical Sciences, College of Pharmacy, Seoul National University, Seoul 151-742, Korea, [c]Department of Biophysics and Chemical Biology, College of Natural Sciences, Seoul National University, Seoul 151-742, Korea, [d]Division of Mass Spectrometry, Korea Basic Science Institute, Ochang-eup Yeongudangiro 162, Cheongwon-gun, Chungbuk 363-883, Korea, [e]Department of Chemistry, Mokpo National University, Chonnam 534-729, Korea, [f]Department of Life Science, Dongguk University-Seoul, Seoul 100-712, Korea

*Corresponding author: S.W.S. (e-mail address: sewonsuh@snu.ac.kr)

Supplementary Figures
Supplementary Tables

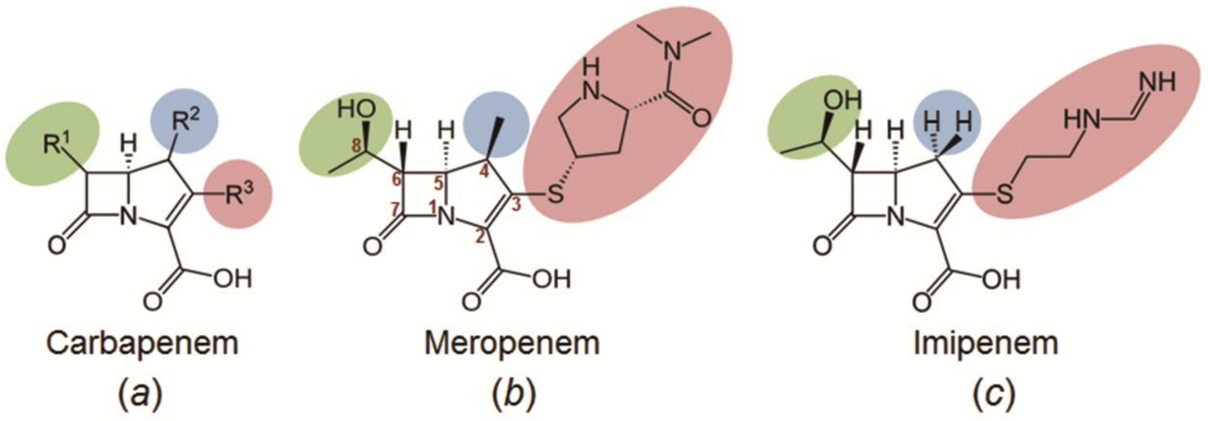

Supplementary Figure S1

Chemical structures of carbapenems. (*a*–*c*) Variable regions (R^1, R^2, and R^3) of carbapenem (*a*), meropenem (*b*), and imipenem (*c*) are shadowed by green, blue, and red, respectively.

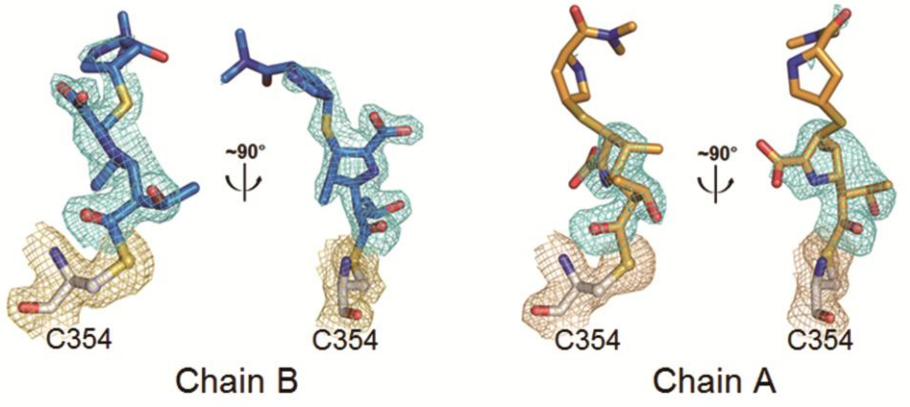

Supplementary Figure S2

Electron density maps of meropenem bound to $Ldt_{Mt2\Delta130}$. Electron density maps of the meropenem adduct covalently bound to Cys354 in chains B and A of the meropenem-complexed $Ldt_{Mt2\Delta130}$. The omit $mF_o - DF_c$ maps (contoured at 2.5σ) for the meropenem adduct and the $2mF_o - DF_c$ maps (contoured at 1.0σ) for Cys354 are colored in blue and yellow, respectively, with their stick models.

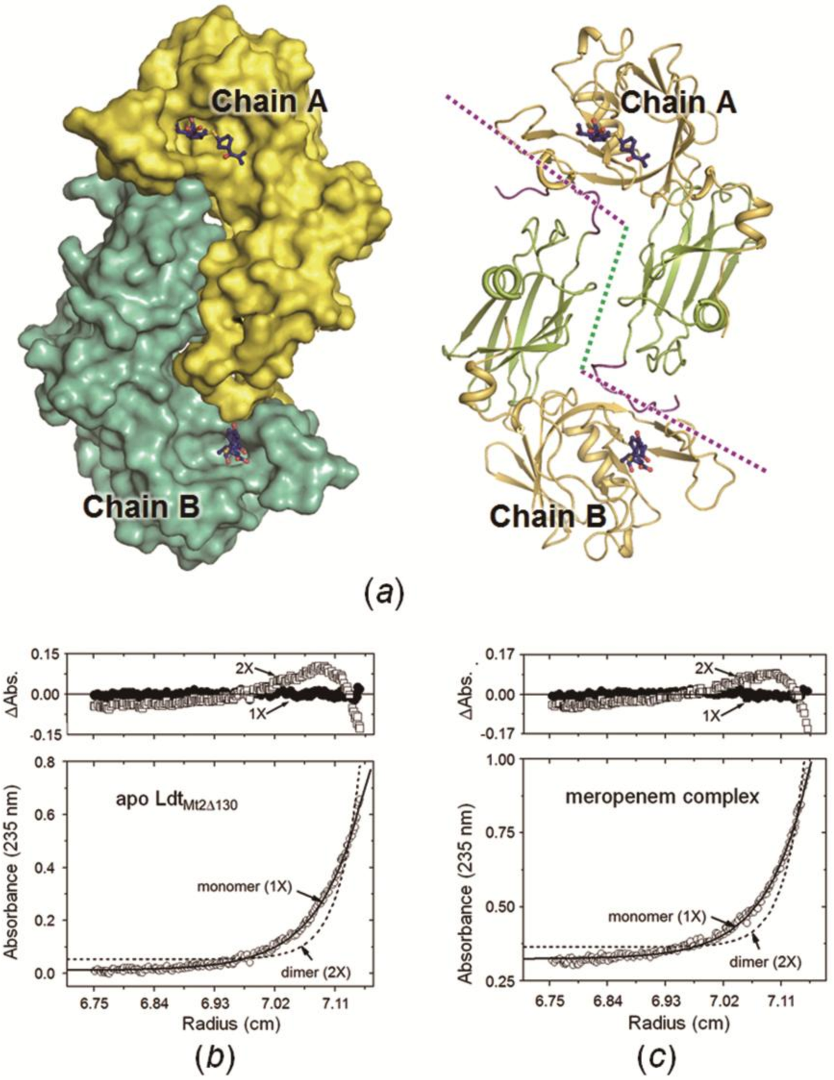

Supplementary Figure S3

Analysis of the oligomeric state of $Ldt_{Mt2\Delta130}$. (*a*) Surface representation and ribbon diagram of two independent monomers of $Ldt_{Mt2\Delta130}$ in the crystal structure of the meropenem-complex, showing a tight interaction between them. (*b*,*c*) Analytical ultracentrifugation data for apo (*b*) and meropenem-complexed $Ldt_{Mt2\Delta130}$ (*c*). These representative data were measured at 235 nm and 24,000 rpm using 1.60 μM protein for both samples. The circles are experimental data and the solid line is a fitting line for an ideal monomer (1x) model. The dotted lines are fitting lines for an ideal dimer (2x) model. Distributions of the residuals for monomer (1x, filled circles) and dimer (2x, square) models are shown in the top panels. Based on the UV spectrum of meropenem-complexed $Ldt_{Mt2\Delta130}$ recorded with XL-A optics, sedimentation data were measured at 235 nm where the effect of meropenem absorption was minimal. Random distributions of the residuals for the monomer (1x) model indicate that

both apo and meropenem-complexed Ldt$_{Mt2\Delta130}$ exist as homogeneous monomers in solution.

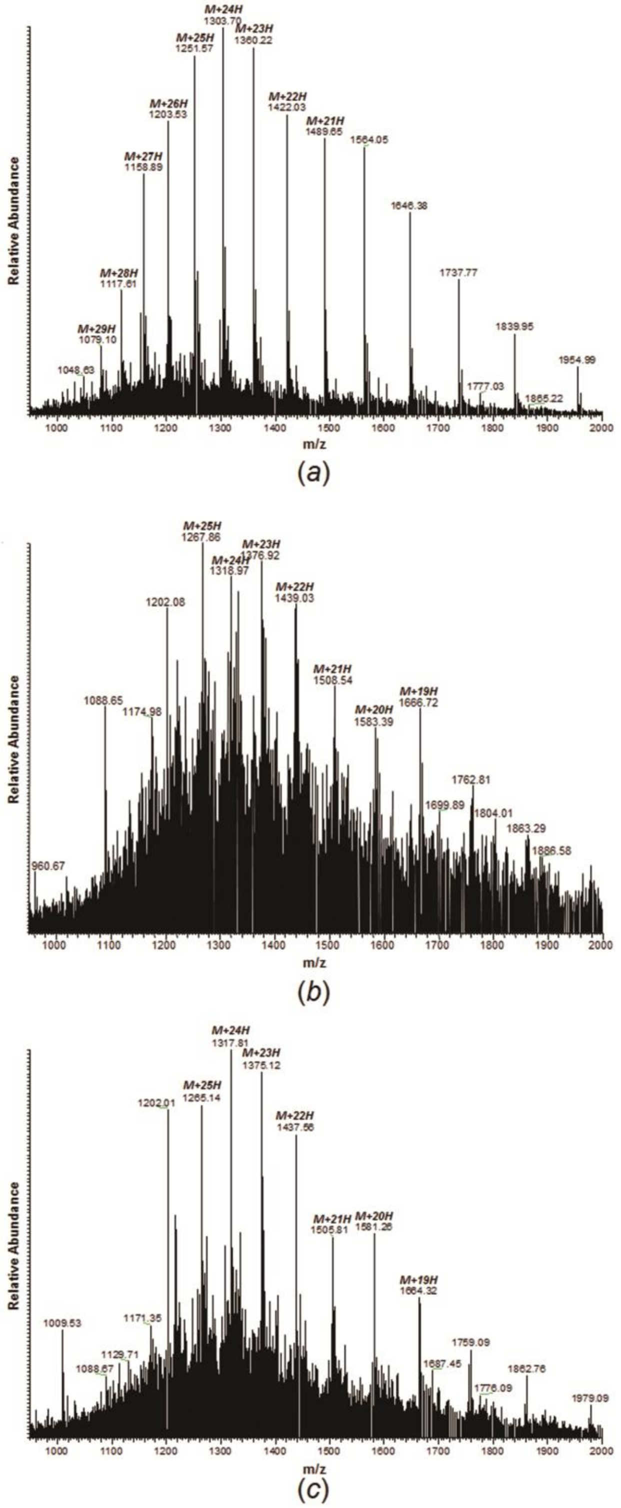

Supplementary Figure S4

Mass spectrometry of Ldt$_{Mt2\Delta130}$. (*a*–*c*) Mass spectra of Ldt$_{Mt2\Delta130}$ in the absence of meropenem (*a*), after 20 min incubation with meropenem (100-fold molar excess) (*b*), and

after three days of incubation with meropenem (*c*) were recorded using a linear ion trap mass spectrometer.

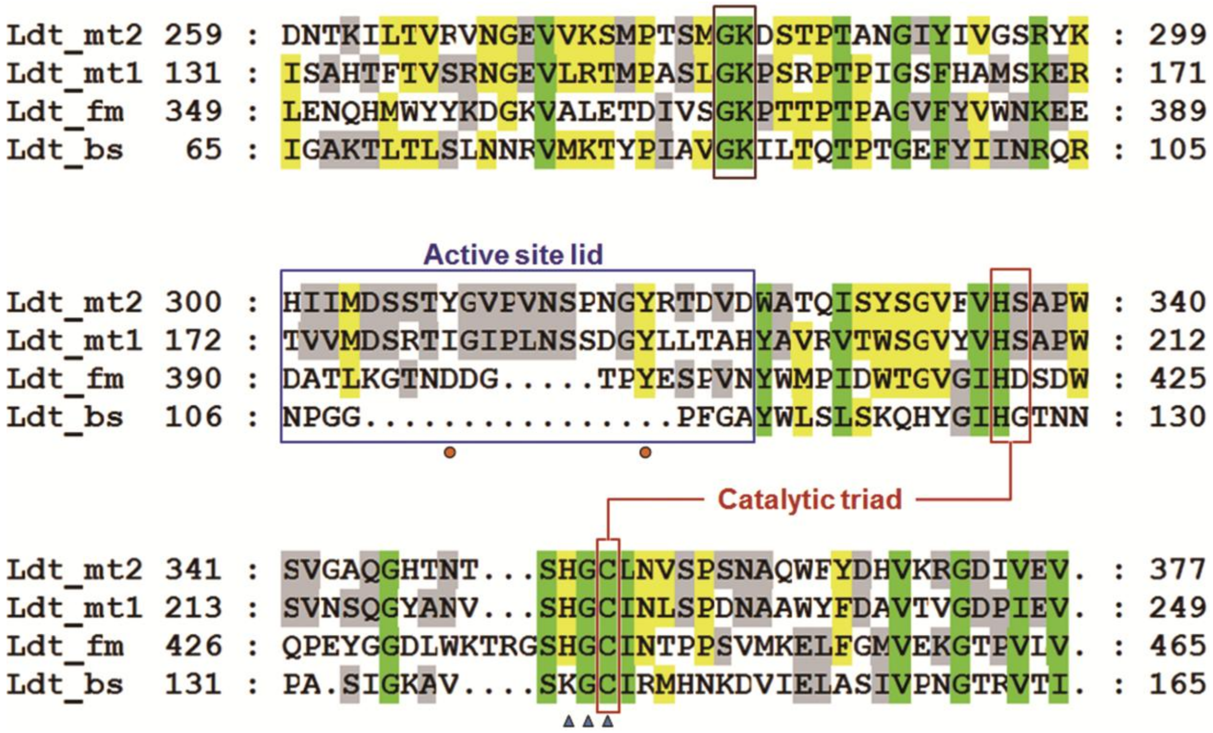

Supplementary Figure S5

Sequence alignment of L,D-transpeptidase domains in four YkuD family proteins. Sequence alignment of *M. tuberculosis* Ldt_{Mt2} (Ldt_mt2; SWISS-PROT accession code: O53223), *M. tuberculosis* Ldt_{Mt1} (Ldt_mt1; O53638), *Enterococcus faecium* Ldt_{fm} (Ldt_fm; Q3Y185), and *Bacillus subtilis* YkuD protein (Ldt_{Bs}) (Ldt_bs; O34816). The catalytic triad (His336, Ser337, and Cys354 in Ldt_{Mt2}) and the active site lid (His300–Asp323 in Ldt_{Mt2}) are enclosed by red and blue boxes, respectively. The residues forming the oxyanion hole (His352, Gly353 and Cys354 in Ldt_{Mt2}) and tyrosines of Ldt_{Mt2} (Tyr308 and Tyr318) that play important roles in the meropenem binding are indicated by blue triangles and orange circles below the sequences, respectively. This figure was drawn with ClustalX (Thompson *et al.*, 1997) and GeneDoc (http://www.nrbsc.org/downloads).

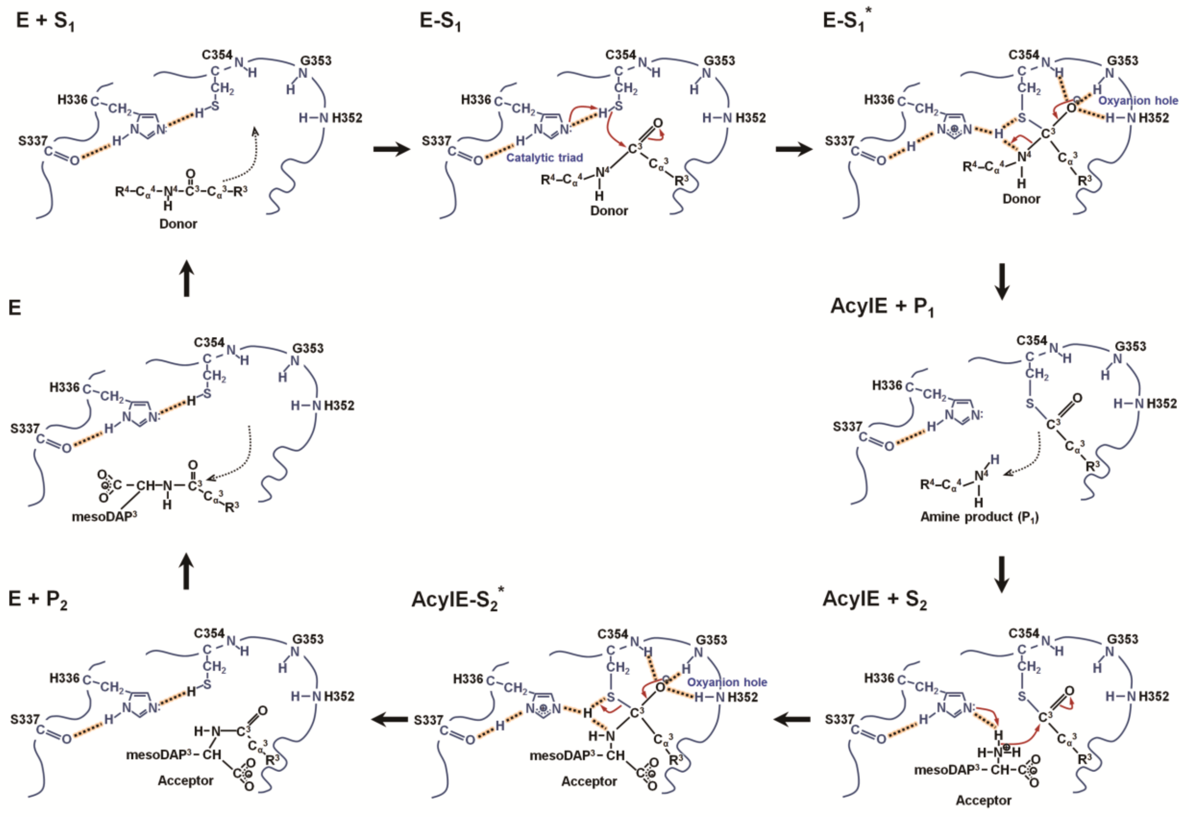

Supplementary Figure S6

A mechanistic model for L,D-transpeptidation by Ldt_{Mt2}. E, Ldt_{Mt2} enzyme; S_1, the donor substrate (meso-DAP^3-D-Ala^4); AcylE, the acylated Ldt_{Mt2} by the S_1 adduct; P_1, the leaving amine product from S_1 after acylation; S_2, the acceptor substrate (meso-DAP^3); P_2, the final product from S_2 by 3→3 cross-linking after transpeptidation. Asterisks denote transition states.

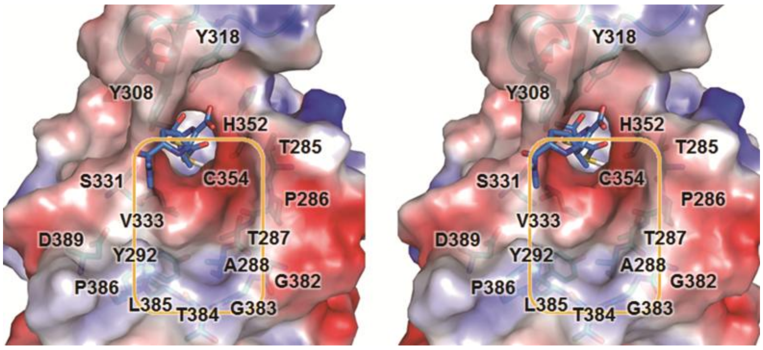

Supplementary Figure S7

A stereo view of the electrostatic potential surface diagram around "Path B" of meropenem-complexed $Ldt_{Mt2\Delta130}$. The surface of the potential binding site for the donor substrate (S_1) or the R^3 side chain of carbapenems is indicated by an orange box (for chain B). The bound meropenem is shown in a stick model.

Supplementary Table S1. Mass analyses of $Ldt_{Mt2\Delta130}$ before and after reacting with meropenem (masses in Da)						
	Theoretical mass (A)	Experimental average mass (B)	Error (B–A)	Experimental meropenem mass[a] (C)	Theoretical mass of intact meropenem (D)	Error (D–C)
Apo	31,246.80	31,263.80	+17.00	-	-	-
Complex (20 min)[b]	31,630.26	31,648.63	+18.37	384.83	383.46	−1.37
Complex (3 days)[c]	31,630.26	31,603.62	−26.64	339.82	383.46	43.64

[a] Experimental meropenem mass (experimental complex mass – experimental apo mass).
[b] Meropenem complex after 20 min reaction.
[c] Meropenem complex after 3 days of reaction.

Supplementary Table S2. Root-mean-square deviations for pair-wise comparisons of $Ldt_{Mt2\Delta130}$ models			
Model	Apo-form	Mercury derivative	Meropenem complex (chain A)
Mercury derivative	0.40 Å for 246 Cα atoms	-	-
Meropenem (chain A)	0.67 Å for 246 Cα atoms	1.34 Å for 263 Cα atoms	-
Meropenem (chain B)	0.77 Å for 246 Cα atoms	1.82 Å for 263 Cα atoms	1.48 Å for 268 Cα atoms

Supplemental Reference

Thompson, J. D., Gibson, T. J., Plewniak, F., Jeanmougin, F. & Higgins, D. G. (1997). *Nucleic Acids Res*. **25**, 4876–4882.
